# Long noncoding RNA regulation of spermatogenesis via the spectrin cytoskeleton in *Drosophila*

**DOI:** 10.1093/g3journal/jkab080

**Published:** 2021-03-15

**Authors:** Mark J Bouska, Hua Bai

**Affiliations:** Department of Genetics, Development, and Cell Biology, Iowa State University, Ames, IA 50011-1079, USA

**Keywords:** lncRNAs, spectrin, actin cap, spermatid nuclear bundles, head cyst cells

## Abstract

The spectrin cytoskeleton has been shown to be critical in diverse processes such as axon development and degeneration, myoblast fusion, and spermatogenesis. Spectrin can be modulated in a tissue specific manner through junctional protein complexes, however, it has not been shown that long noncoding RNAs (lncRNAs) interact with and modulate spectrin. Here, we provide evidence of a lncRNA CR45362 that interacts with α-Spectrin, is required for spermatid nuclear bundling during *Drosophila* spermatogenesis. We observed that CR45362 showed high expression in the cyst cells at the basal testis, and CRISPR-mediated knockout of *CR45362* led to sterile male, unbundled spermatid nuclei, and disrupted actin cones. Through chromatin isolation by RNA precipitation—mass spectrometry (ChIRP-MS), we identified actin-spectrin cytoskeletal components physically interact with the lncRNA CR45362. Genetic screening on identified cytoskeletal factors revealed that cyst cell-specific knockdown of α-Spectrin phenocopied *CR45362* mutants and resulted in spermatid nuclear bundle defects. Consistently, *CR45362* knockout disrupted the co-localization of α-Spectrin and spermatid nuclear bundles in the head cyst cells at the basal testis. Thus, we uncovered a novel lncRNA CR45362 that interacts with α-Spectrin to stabilize spermatid nuclear bundles during spermatid maturation.

## Introduction

Long noncoding RNAs (lncRNAs) comprise roughly half of the human genome (Hrdlickova *et al.* 2014; Hon *et al.* 2017; Pertea *et al.* 2018) and constitute a pool of largely unexplored modulatory molecules that can regulate cytoskeletal proteins, endosomes, and autophagic pathways in disease (Xu *et al.* 2017; Krause 2018; Tang *et al.* 2018). LncRNAs often act in a tissue specific manner (Jandura and Krause 2017), in particular, they are developmentally significant as a large number of lncRNAs demonstrate high expression in the testis in wide-ranging species from *Drosophila*, to mice, to humans (Wen *et al.* 2016; Tang *et al.* 2017; Zhang *et al.* 2017; 2019). The testis specificity of lncRNAs has been posited as a possible mechanism by which increased organism complexity occurs through gain of function in other tissues (Jandura and Krause 2017). This specificity also offers a unique opportunity to identify targets for male nonhormonal contraceptives and identify underlying causes of idiopathic male infertility, but few lncRNAs have been mechanistically pursued in this context.

During mammalian sperm maturation, Sertoli cells surround developing sperm and undergo intricate endocytic exchanges of intercellular junctions (ectoplasmic specialization) and tubulobulbar complexes (actin-spectrin-clathrin structures) to facilitate the movement of spermatocytes towards the lumen of the seminiferous tubules and hold the spermatid until mature, while maintaining the blood-testis barrier (Aristaeus de Asis *et al.* 2013). In *Drosophila* the male germ cells are encompassed and maintained by 2 Cyst cells, which are similar to human Sertoli cells (Zoller and Schulz 2012). The cyst cells provide molecular cues and provide protection until sperm maturate and are released into the seminal vesicle (Leatherman and Dinardo 2010). Spermatogenesis requires dynamic remodeling of the actin-spectrin cytoskeleton in maturating sperm and cyst cells as the 64 post-meiotic sperm elongate their tails, condense their nuclei forming tight thin structures that are held in a bundle within the cyst cells as the cyst cells in turn have to traverse the length of the testis (Ghosh-Roy *et al.* 2004; Dubey *et al.* 2016; Dunleavy *et al.* 2019). The head cyst cell (HCC) then attaches to terminal epithelial cells in the basal testis and the sperm tails coil, to orient the spermatid tails towards the seminal vesicle for release (Dubey *et al.* 2016). Although endosomal regulation of junctional complexes (Dubey *et al.* 2019; Liu *et al.* 2020) and actin dynamics (Noguchi *et al.* 2008; Desai *et al.* 2009) play major roles in *Drosophila* spermatogenesis, how the complex cytoskeletal remodeling processes are tightly controlled in each interacting cell type during *Drosophila* spermatogenesis is still not completely understood.

Herein, we identify an uncharacterized lncRNA *CR45362* in *Drosophila* that interacts with α-Spectrin to stabilize spermatid nuclear bundles in the terminal epithelium region of basal testis. The spectrin cytoskeleton is modulated in axon development and degeneration (Wang *et al.* 2018; 2019), myoblast fusion (Duan *et al.* 2018), and spermatogenesis (Aristaeus de Asis *et al.* 2013) through junctional protein complexes (Machnicka *et al.* 2019), We found that *CR45362* knockout results in male sterility, disrupted spermatid nuclear bundling and interaction between α-Spectrin and actin cap. Our results indicate a novel mechanism by which the spectrin cytoskeleton can be contextually regulated through lncRNAs.

## Materials and methods

### *Drosophila* husbandry and strains

A list of fly strains is provided in Supplementary Table S1. Flies were maintained at 25 ^°^C, 60% relative humidity, and 12-hour light/dark cycle. Adults and larvae were reared on a standard cornmeal and yeast-based diet, unless otherwise noted. The standard cornmeal diet consists of the following materials: 0.8% cornmeal, 10% sugar, and 2.5% yeast.

### Generation of CR45362 knockout lines

Knockouts were generated using previously described protocols (Gratz *et al.* 2014). Two CRISPR gRNAs were designed using CRISPR Optimal Target Finder, https://flycrispr.org/target-finder. pU6-2-gRNA plasmids (*Drosophila* Genomics Resource Center stock# 1363) were cut with BBSI-HF restriction enzyme (Fisher Scientific Catalog# NC1222468) in 1X CutSmart Buffer and de-phosphorylated with Calf Intestinal Alkaline Phosphatase (Fisher# 50811712). gRNA oligos (CTTCGGCTTGCAAAGGGGGTATGT and AAACGTCTATCTGTTGGTTTTCCC) were annealed and ligated with linearized pU6-2-gRNA plasmids using T4 DNA ligase (New England Biolabs # M0202S). Plasmids containing the gRNAs were then amplified and verified by Sanger sequencing. Two gRNA plasmids were co-injected into fly embryos expressing germ line-specific Cas9 protein (Bloomington #51324). Injection service was provided by Rainbow Transgenic Flies Inc. The CR45362 deletion was selected and maintained by crossing in a Cyo balancer. PCR primers used in deletion verification were: TATACTCGGCGCTCCTCTCA and ACGAGTGCAGACCGAAAACA.

### LysoTracker assay

To induce starvation response 3–5-day old male flies were put on 1 ml of 1X PBS (Life Technologies # 10010-023) added to a Kimwipe in vial overnight for 12–16 hours. Fat bodies were dissected in 1X PBS then incubated in Lysotracker (Invitrogen # L7528) and Hoechst 33342 (Immunochemistry Technologies# 639) according to manufacturer’s specifications. One replicate consists of the average of three 10 μm diameter circles per fly fat body measured by CellSens software for percentage of area containing fluorescence, with more than three flies examined for each genotype.

### Fertility assay

Individual virgin females were crossed with individual males and mated in food vials with added dry yeast. The numbers of adult progeny were recorded within 4–5 days of eclosion.

### Fluorescence *in situ* hybridization

RNA from WT 3–5-day old flies was extracted and reverse transcribed to cDNA using Power SYBR Green Cells-to-Ct Kit (Invitrogen# 4402953) following manufacturer’s protocol. CR45362 template was generated following manufacturers protocol by PCR Amplification using DreamTaq DNA Polymerase with buffer (Thermo Scientific# EP0701), 10 mM dNTPs (Sigma Aldrich# DNTP100-KT), 5 pmol/ul custom primers (GTCGGGACGGAATGAGAGTG and GAGCGAGCCACTTGAGCATA), and ∼1 ug of total cDNA from above. Products were then ligated into pGEM-T Easy Vector (Promega# A1360). Plasmids were linearized for fluorescent probe production using Invitrogen FISH tag RNA Red Kit (Invitrogen# F32954). Dissected testis was incubated with fluorescent probes following manufacturer’s protocol, and imaged with an epifluorescence-equipped Olympus BX51WI microscope.

### Fluorescent immunohistochemistry

The following protocols are dependent on antibodies and samples. Testes from 3- to 5-day old flies were dissected in 1X PBS, fixed in 4% paraformaldehyde diluted in 0.1–0.3% PBST (1X PBS plus Triton X-100, Fisher# BP151-500) for 20–30 minutes at room temperature. Samples were washed and optionally blocked with PBST + BSA (0.25 g Bovine Serum Albumin (Sigma Aldrich# A7906-500G) per 50 ml PBST) three times, 10 minutes each. The samples were incubated with primary antibodies overnight at 4 °C or for 2 hours at room temperature. Samples were next washed in PBST 3 times for 10 minutes each wash, then incubated with secondary antibodies for 2 hours at room temperature. Samples were again washed in PBST 3 times for 5 minutes each. For F-actin staining, samples were co-stained with Hoechst (Immunochemistry Technologies# 639) and Alexa Fluor Phalloidin (Thermo Fisher scientific# A12381) for 30 minutes followed by one wash in 1X PBS and mounted in ProLong Diamond Antifade Mountant (Invitrogen# P36961). Primary antibodies are as follows; Fasciclin III (DSHB # 7G10) (Dubreuil *et al.* 1987; Patel *et al.* 1987), α-Spectrin (gifts from Dr. Ronald Dubreuil at UIC) (Dubreuil *et al.* 1987; Diaconeasa *et al.* 2013), γ-Tubulin (1:500) (Sigma Aldrich# T5326). Secondary antibodies are as follows: Alexa Fluor 488 anti-rabbit IgG (Jackson ImmunoResearch # 711-545-152), Alexa Fluor 594 anti-rabbit IgG (Jackson ImmunoResearch # 711-585-152), Alexa Fluor 488 anti-mouse IgG (Jackson ImmunoResearch # 115-545-166), Alexa Fluor 594 anti-mouse IgG (Jackson ImmunoResearch # 115-585-166).

### Microscopy and image analysis

Images taken from Olympus BX51WI fluorescent microscope and FV3000 confocal microscope were processed with CellSens Software and finalized using ImageJ Fiji. Phase contrast images were taken on a Zeiss Axioskop phase contrast microscope capture with ProgRes MAC CapturePro 2.7.

### Chromatin isolation by RNA precipitation—mass spectrometry

ChIRP-MS was performed following published protocols (Chu and Chang 2018). Antisense DNA oligos (even or odd tiling of probes) were designed using Bioresearch Technologies ChIRP Probe Designer. Probe sequences were listed in Supplementary Table S2. About 547∼662 testis were dissected from 3- to 5-day old flies, 547 testis of CR45362 KO, 569 testis of WT (even probe), and 662 testis of WT (odd probe). Probes corresponding to the deleted segment were used as a control. Testis were dissected in ice cold RNase free 1X PBS. Dissection tools and surfaces were cleaned by RNaseZap RNase Decontamination Solution (Ambion# 9780). Testis were fixed in 3% paraformaldehyde followed by quench with 2.5 M glycine (Sigma Aldrich# 410225-50 G). Biotinylated probes and streptavidin beads (Dynabeads™ MyOne™ Streptavidin C1, Thermo #65001) were used to pull down proteins bound to CR45362 (Figure 5A). Mass spectrometry was performed by the Taplin Biological Mass Spectrometry Facility at Harvard University. The number of identified peptides were normalized to the total number of testis of each sample. STRING-db (https://string-db.org) was used for Gene Ontology (GO) analysis.

### Genetic RNAi screen

Five pairs of testis were dissected from 3- to 5-day old males for each RNAi cross. DAPI nuclear staining was used to screen for spermatid nuclear dispersion phenotype. Gal4 tissue driver and RNAi lines can be found in Supplementary Table S1.

### Statistical analysis

GraphPad Prism (GraphPad Software, version 6.07) was used for statistical analysis. Unpaired two-tailed Student’s *t*-test and two-way ANOVA were performed for LysoTracker analysis.

### Data availability

Strains and plasmids are available upon request. The authors affirm that all data necessary for confirming the conclusions of the article are present within the article, figures, and tables. Supplementary material is available at figshare: https://doi.org/10.25387/g3.14183207.

## Results

### Deletion of lncRNA *CR45362* leads to male infertility

**Figure 1:**
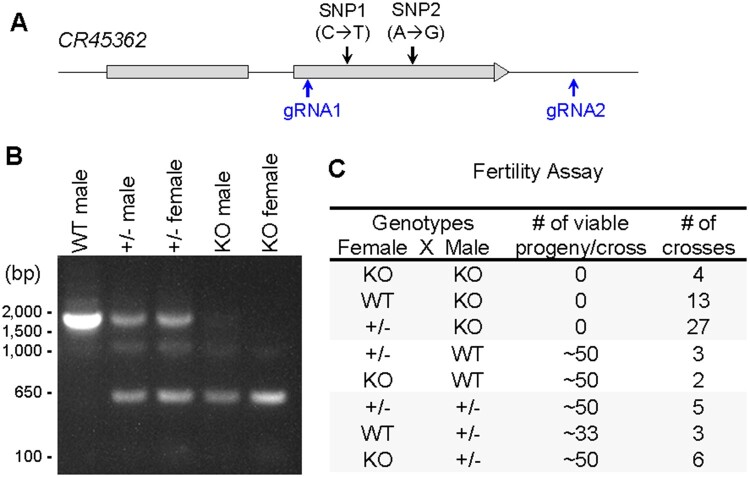
Deletion of lncRNA *CR45362* leads to male fertility. (A) Diagram showing *CR45362* locus and the locations of two guide RNAs and SNPs identified in GWA analysis. (B) PCR analysis confirming the deletion of *CR45362*. Primers are flanking the targeted deletion region. (C) Fertility was tested by crossing individual virgin females with individual males. The numbers of viable adult progeny are recorded within 4–5 days of eclosion. WT: *w^1118^*, ±: heterozygous *CR45362* deletion, KO: homozygous *CR45362* deletion.

In a recent genome-wide association (GWA) analysis on autophagy regulation using the *Drosophila* Genetic Reference Panel (DGRP) (Mackay *et al.* 2012), we identified 360 unique single nucleotide polymorphisms (SNPs) that are significantly associated with variation in basal autophagy-lysosome activity (unpublished). Besides those SNPs associated with protein-coding genes, we found that two SNPs were located in the second extron of an uncharacterized lncRNA CR45362 (Figure 1A). To study the function of lncRNA CR45362 in *Drosophila*, we generated a deletion mutant (*CR45362^KO^* or *KO*) using CRISPR/Cas9 (see materials and methods). The deletion of second extron of *CR45362* was confirmed through PCR (Figure 1B) and Sanger sequencing. The deletion mutants developed normally during the larval and pupal stages (data not shown), however, they exhibited reduced lysosome activities in adult fat body (Supplementary Figure S1). This finding confirmed our GWA analysis. Surprisingly, besides impaired lysosomal function, we also uncovered a male fertility defect associated with the deletion mutants (Figure 1C). We found that the crosses with male homozygous *CR45362^KO^* were unable to produce viable offspring (Figure 1C), suggesting that CR45362 might be involved in the regulation of male fertility.

### Deletion of *CR45362* results in a loss of spermatid nuclear bundling

**Figure 2:**
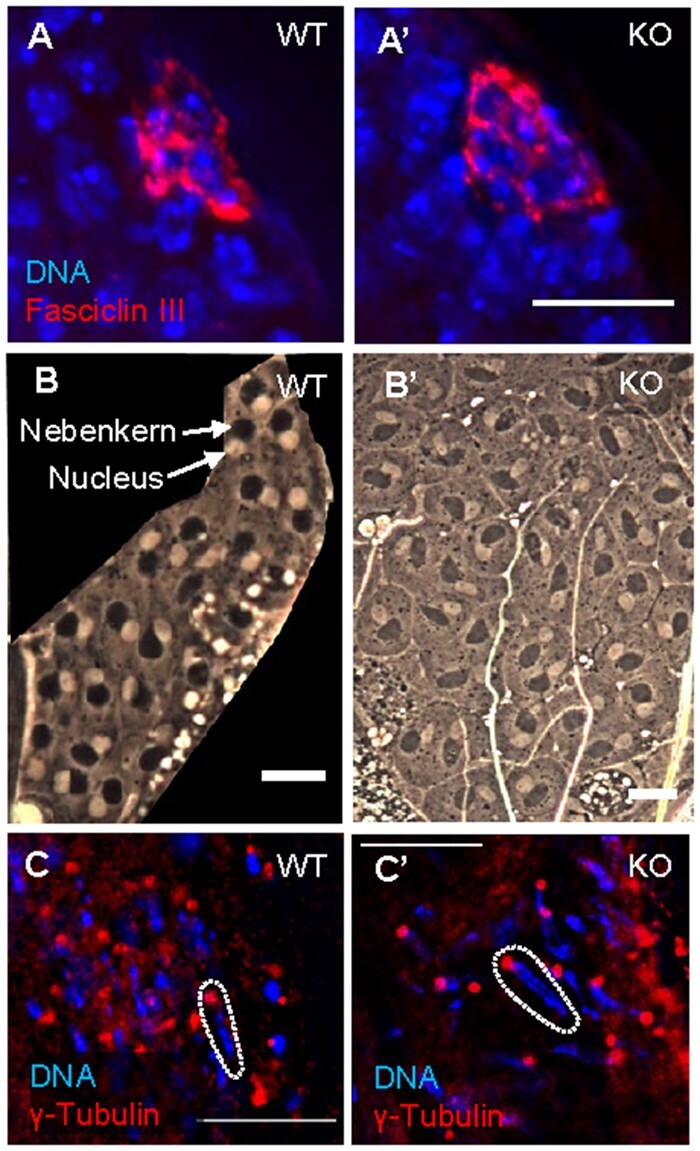
CR45362 deletion does not affect early spermatogenesis processes. (A–A’) Hub cells (indicated by Fasciclin III) of WT and KO testis have proper radial arrangement and position at the apical tip. (B–B’) Phase-contrast imaging of WT and KO spermatids shows a 1:1 ratio of nucleus to nebenkern. (C–C’) Proper centriolar adjunct marked by γ-tubulin in both WT and KO. Dotted lines highlight a single nuclei with properly attached basal body. Scale bars: 10 μm. Three to five biological replicates. All replicates are consistent with the image shown.

Although *CR45362* deletion altered lysosome activity in fat body (Supplementary Figure S1), we did not observe major lysosome defects in mutant testis (data not shown). In order to determine the point at which *CR45362* deletion was causing the male fertility defect, we examined the major processes known to disrupt spermatogenesis (Lifschytz and Hareven 1977; Fabrizio *et al.* 1998; Steinhauer 2015). We used immunostaining or phase-contrast imaging to examine the hub cells (Figure 2, A and A’), round stage spermatids with nebenkern (Figure 2, B and B’), and canoe stage spermatids (Figure 2, C and C’). We found that deletion of *CR45362* did not alter the radial arrangement and position of hub cells (indicated by Fasciclin III). In addition, wild-type (WT) and KO spermatids show correct 1:1 ratio of nucleus to nebenkern (Figure 2, B and B’) and proper centriolar adjunct structure marked by γ-tubulin (Figure 2, C and C’). Our imaging analysis showed that CR45362 knockout did not alter above structures that are associated with early spermatogenesis processes.

**Figure 3:**
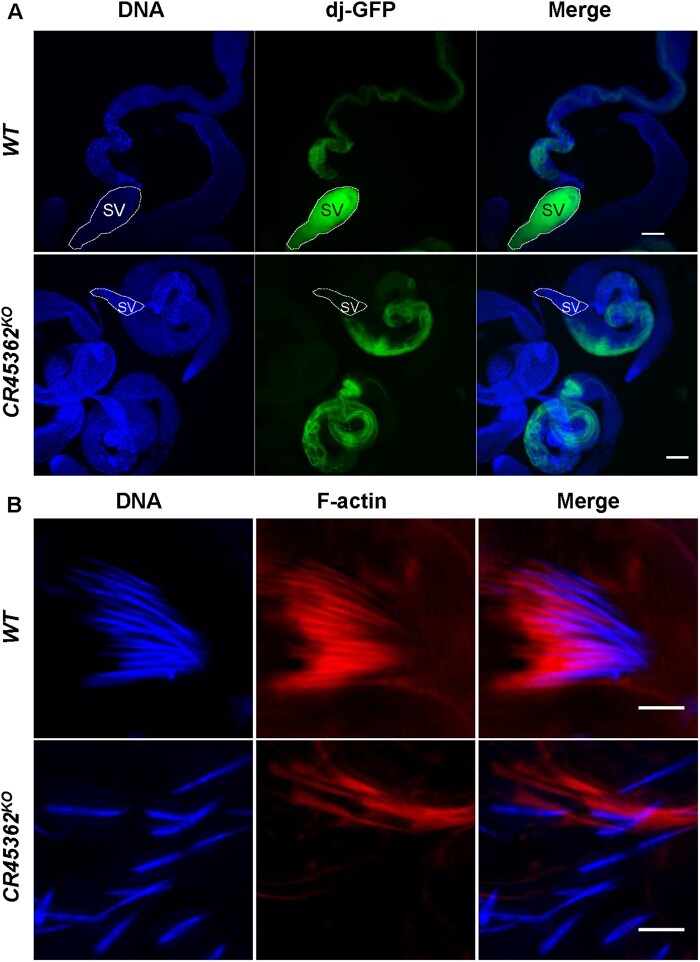
Deletion of *CR45362* results in a loss of spermatid nuclear bundling. (A) Fluorescence imaging shows that spermatid tails marked by *dj-GFP* form properly, but sperms from KO males fail to enter the seminal vesicle (SV). Scale bar: 100 μm. (B) Confocal images of spermatid nuclei from both WT and KO testis. *CR45362* deletion disrupts nucleus bundle and actin cones (indicated via phalloidin staining). Scale bar: 5 μm. Three to five biological replicates. All replicates are consistent with the image shown.

We then crossed *CR45362^KO^* into a *dj-GFP* line to visualize sperms in the mutants. Although sperm tails were fully developed, sperm of *CR45362* knockout mutants were unable to enter the seminal vesicle (Figure 3A). An important step during late stages of spermatogenesis in *Drosophila* is individualization, a process where a cyst of 64 spermatids elongates and matured into individual sperm (Fabrizio *et al.* 1998). During the individualization process, actin cones (labeled by F-actin) assemble around the spermatid, and then move down the length of the spermatids and remove cytoplasmic contents. In *CR45362^KO^* mutants, spermatid nuclei were elongated (Figure 3B), however, the elongated spermatid nuclei appeared dispersed and were not bundled together. In addition, the actin cones of the individualization complex were not properly formed in *CR45362 ^KO^* mutants (Figure 3B). These findings suggest that CR45362 may be involved in individualization process, especially spermatid nuclear bundling.

### The lncRNA CR45362 localizes to the terminal epithelium region of testis and interacts with cytoskeletal proteins

**Figure 4:**
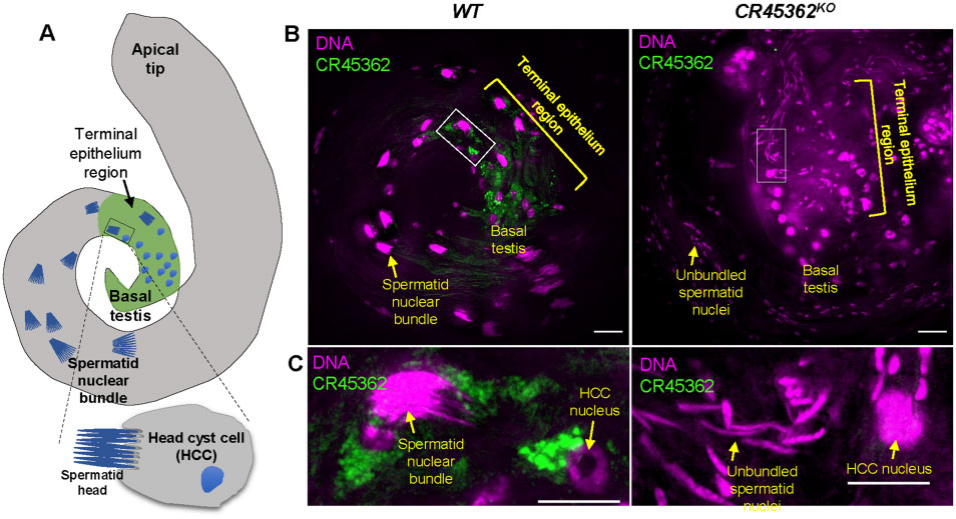
The lncRNA CR45362 localizes to the terminal epithelium region of testis. (A) Schematic diagram showing testis structure. Elongated spermatid nuclei are bundled and moving toward the terminal epithelium region, where the spermatid head is enclosed within the membrane of HCC. (B) FISH showing that lncRNA CR45362 localizes to the terminal epithelium region, nearby spermatid head, and HCC nuclei. No expression of CR45362 was detected in the knockout testis. Scale bar: 20 μm. (C) Enlarged inset from (B). Scale bar: 100 μm. Three to five biological replicates. All replicates are consistent with the image shown.

LncRNAs function in the nucleus, cytoplasm, or even extracellularly, thus, establishing the cellular localization of a lncRNA of interest is a critical step when attempting to determine its function (Kung *et al.* 2013; Cao *et al.* 2018). Due to the strong sterility phenotype seen in CR45362 mutants, we performed fluorescence *in situ* hybridization (FISH) to examine the expression of *CR45362* in *Drosophila* testis. As shown in Figure 4, CR45362 had high expression at the terminal epithelium region of basal testis. It localizes in the cytoplasm of HCCs that surround bundles of elongated spermatid nuclei (Figure 4). The subcellular localization of CR45362 suggests that it may be involved in spermatid-HCC interaction and spermatid maturation. The expression CR45362 was not detected in the knockout testis (Figure 4).

**Figure 5:**
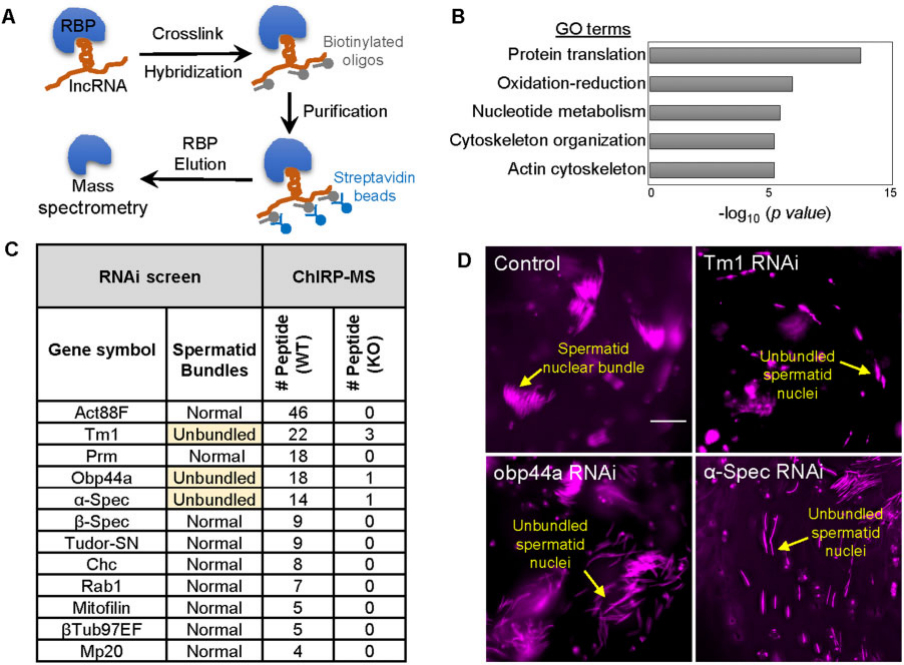
ChIRP-MS and Genetic screen identify candidate proteins involved in CR45362-regulated spermatid nuclear bundle formation. (A) Diagram showing the procedures of ChIRP-MS. (B) GO analysis for the top CR45362 binding partners identified through ChIRP-MS. (C) RNAi screen summary of 12 ChIRP-MS candidate proteins. Five pairs of testis from 3- to 5-days-old males from each knockdown flies were imaged and examined for spermatid bundling. Two rounds of screening (10 testis per round) are performed. At least 6 out of 10 show nuclear bundling defective. Cyst cell driver *Ptc-Gal4* is used to drive gene knockdown in HCC. Control genotype is *Ptc-Gal4 >UAS-GFP*. The numbers of peptide identification of each candidate protein from ChIRP-MS analysis are shown in the last two columns (see Supplementary Table S3 for more information). (D) Confocal images showing spermatid nuclear bundle defects in testis dissected from *Tm1 RNAi*, *Obp44a RNAi*, and *α-Spec RNAi* flies. DAPI is used for nucleus staining. Scale bar: 20 μm.

The cytoplasmic localization of CR45362 suggested a potential interaction between this lncRNA and cytoplasmic proteins. Therefore, to determine the protein binding partners for CR45362 we performed Chromatin Isolation by RNA Precipitation—Mass Spectrometry (ChIRP-MS) on dissected testes from 3- to 5-day-old males. ChIRP-MS utilizes streptavidin magnetic bead and biotinylated probes complementary to the lncRNA to pull down lncRNA-RNA binding protein complex (Figure 5A). Mass-spectrometry was then performed to identify candidate proteins that bind to the lncRNA. About 232 proteins show twofold higher detection in WT ChIRP samples than KO (Supplementary Table S3). Many of them showed no detection in KO. GO analysis showed an enrichment of pathways like protein translation, oxidation-reduction, nucleotide metabolism, and cytoskeleton organization (Figure 5B). Many key cytoskeleton proteins, such as Act88F, Tropomyosin 1/Tm1, alpha-Spectrin/α-Spec, and beta-Spectrin/β-Spec, are among the top hits (Figure 5C). The actin cytoskeleton is known to play a vital role in spermatogenesis and male fertility (Dunleavy *et al.* 2019), including forming the actin cap structure to hold spermatids around the HCC membrane (Dubey *et al.* 2016). Given that CR45362 localizes in HCC surrounding spermatid bundles (Figure 4), it is likely that CR45362 interacts with cytoskeletal proteins to facilitate HCC-spermatid interaction.

### lncRNA CR45362 interacts with α-Spectrin to stabilize spermatid nuclear bundles

To genetically examine which candidate partner proteins may be involved in CR45362-regulated spermatid nuclear bundle formation, we performed a RNAi screen on twelve CR45362-interacting proteins identified from our ChIRP-MS analysis (Figure 5C). A cyst cell driver *Ptc-Gal4* was used in the screening. We have verified the expression of this driver in the cyst cells located in the terminal epithelium region of basal testis (Supplementary Figure S2), similar to where CR45362 is expressed (Figure 4). Our genetic screen revealed that cyst cell-specific knockdown of three candidates, *Tm1*, *Obp44a*, and *α-Spec*, lead to severe defects in spermatid nuclear bundling (Figure 5D). Surprisingly, knockdown of Act88F did not alter spermatid nuclear bundling, which is probably due to compensatory mechanisms through the activation of other actin isoforms (Perrin and Ervasti 2010; Figure 5C). Both Tm1 and α-Spec are important components in the actin-spectrin cytoskeleton complex and play important roles in spermatogenesis (Texada *et al.* 2011; Dunleavy *et al.* 2019). It is known that α-Spectrin plays crucial role in the formation of spermatocyte fusomes (Schulz *et al.* 2004), however, its role in spermatid nuclear bundling and HCC-spermatid interaction is less understood. Interestingly, we found that cyst cell-specific knockdown of *α-Spec* did not affect fusome formation in the apical tip of testis (Supplementary Figure S3). Thus, our results suggest that α-Spectrin expression in the cyst cells at the basal testis may play specific role in stabilizing spermatid nuclear bundles, probably through the interaction with other cytoskeletal components and formation of spectrin junctional complexes.

**Figure 6:**
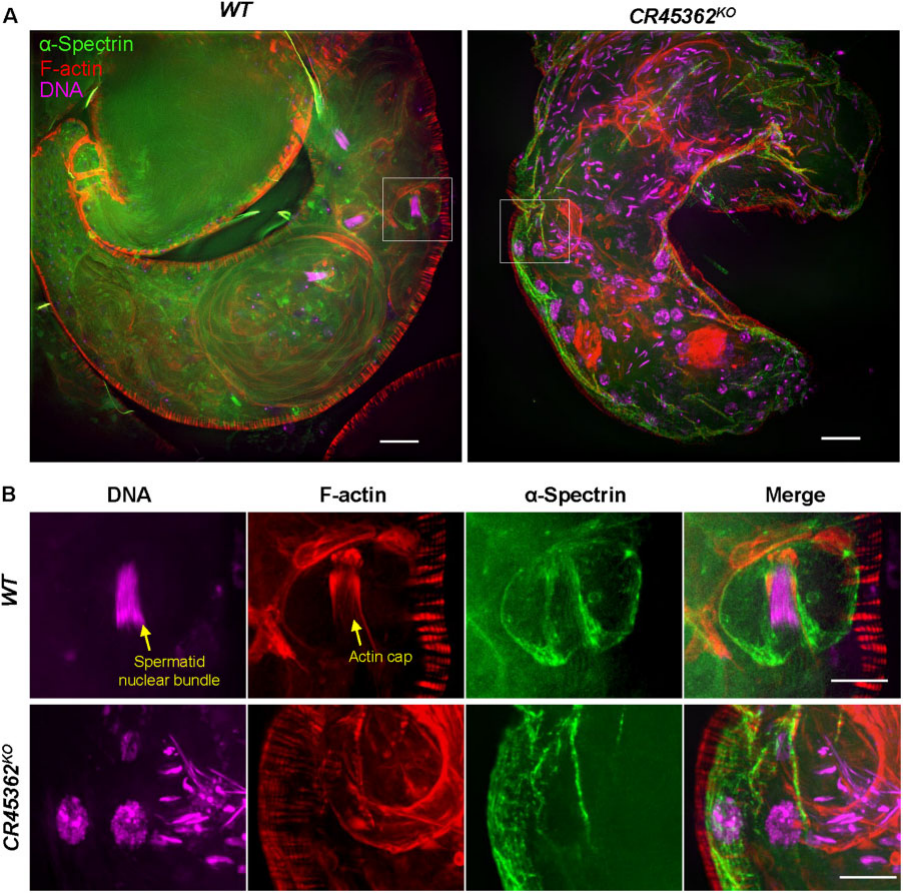
*CR45362* knockout disrupts the interaction between α-Spectrin and actin cap. (A) Confocal images showing the expression pattern of α-Spectrin at the terminal epithelium region of testis in both wild-type and *CR45362* knockout. Scale bar: 20 μm. (B) Zoom-in image of the white insets from (A). Scale bar: 10 μm. Three to five biological replicates. All replicates are consistent with the image shown.

Our ChIRP data and genetic screen implicated that spectrin cytoskeletal complexes might be involved in CR45362-regulated spermatid nuclear bundling. One might predict that CR45362 directly binds α-Spectrin and promotes the interaction between α-Spectrin and spermatid nuclear bundles. To test this idea, we performed immunostaining to monitor α-Spectrin subcellular localization in *CR45362* mutants. Interestingly, α-Spectrin was found in HCC of the terminal epithelium region and it was co-localized with the actin cap structure surrounding spermatid nuclear bundles (Figure 6, A and B). The actin cap is important in holding the spermatid heads and preventing their premature release (Dubey *et al.* 2016). Consistent with our prediction, *CR45362* knockout disrupted the co-localization between α-Spectrin and spermatid heads, as well as the interaction between α-Spectrin and the actin cap (Figure 6, A and B). These results suggest that CR45362 interacts with α-Spectrin to stabilize spermatid nuclear bundles.

Recently, a proteomic study identified 1,174 binding sites within 529 proteins in HeLa cell (Castello *et al.* 2016). In this study, seven noncanonical RNA-binding domains were identified for human α-spectrin nonerythrocytic 1 (SPTAN1), the homolog of *Drosophila* α-spectrin (Supplementary Table S4). We performed a sequence alignment analysis and found that the RNA-binding domains of human SPTAN1 share 55-90% sequence homology with *Drosophila* α-Spectrin (Supplementary Table S4). Thus, it is likely that *Drosophila* α-Spectrin may be able to directly interact with RNAs (like CR45362) via these RNA-binding domains.

## Discussion

In this study, we have demonstrated that the *Drosophila* lncRNA CR45362 localizes to the terminal epithelium region of testis and plays a key role in regulating spermatid nuclear bundles through interacting with cytoskeletal component α-Spectrin. Mutation of *CR45362* results in male sterility, loss of spermatic nuclear bundles, and disrupted α-Spectrin and actin cap interaction.

The loss of α-Spectrin throughout the somatic cells in *CR45362^KO^* testis is possibly due to the lack of proper force generation or membrane deformability that would allow the cyst membrane to sense and fold around a bundle of spermatid nuclei. It has been shown that dispersed α/βH-Spectrin heterotetramers are generated once the forces are removed in myoblast fusion (Duan *et al.* 2018). The force from myoblast invasion triggers the interaction between α-Spectrin and βH-Spectrin during the process of myoblast fusion (Duan *et al.* 2018). Our preliminary study also showed that βH-Spectrin accumulated at the site of spermatid penetration into the cyst, whereas *CR45362* knockout disrupted βH-Spectrin accumulation and its interaction with α-Spectrin.

The high expression levels of lncRNAs in the testes (Soumillon *et al.* 2013; Necsulea *et al.* 2014; Tang *et al.* 2017) have been posited as a possible mechanism by which increased organism complexity occurs through gain of function in other tissues (Jandura and Krause 2017). This aligns well with the correlation of the increasing number of lncRNAs with increasing organism complexity (Jandura and Krause 2017). Adding to this reasoning, we demonstrate that lncRNAs are able to support structural tasks in cells and that lncRNAs may play a significant role in the modulation of cytoskeletal structures which are also known to contribute to the increasing tissue complexity of higher organisms (Huber *et al.* 2013). LncRNAs have been identified as modulating actin and intermediate filament cytoskeletal components in cancer cell cultures (Tang *et al.* 2018), and our finding further extends this overall lncRNA-cytoskeletal framework.

This work provides unique data indicating that the spectrin structures in the testes can be organized by lncRNA. This creates several new questions that provide interesting opportunities for future work. First, is spectrin regulated by lncRNAs during mammalian spermatogenesis, and what RNA motifs regulate their interaction? Does the spectrin cytoskeleton have a diversity of tissue specific regulatory lncRNAs, and how do these lncRNAs influence spectrin-regulated processes in those tissues?
